# Special Issue “Advances in Monitoring Metabolic Activities of Microorganisms by Calorimetry”

**DOI:** 10.3390/microorganisms11051204

**Published:** 2023-05-04

**Authors:** Daumantas Matulis, Lars Wadsö, Karim Fahmy

**Affiliations:** 1 Life Sciences Center, Vilnius University, Saulėtekio al. 7, 10257 Vilnius, Lithuania; daumantas.matulis@bti.vu.lt; 2 Division of Building Materials, Lund University, Box 118, 221 00 Lund, Sweden; lars.wadso@byggtek.lth.se; 3 Department of Biophysics, Helmholtz-Zentrum Dresden-Rossendorf, Bautzner Landstrasse 400, 01328 Dresden, Germany; 4 Cluster of Excellence Physics of Life, Technische Universität Dresden, Arnoldstraße 18, 01307 Dresden, Germany

In recent decades, the calorimetric monitoring of microbial metabolism, i.e., the time-dependent measurement of metabolic heat, has profited from technical progress that rendered isothermal microcalorimetry (IMC) not only more sensitive, but also easier to perform in the non-specialist lab using commercial equipment [1]. In combination with the low demand for preparatory work and man-power, as well as direct access to thermodynamic state variables, this has led to the broader use of this method in microbiological, environmental, and medical studies. With IMC, as well as with isothermal titration calorimetry (ITC), heat production curves are obtained which report enthalpies and entropies of ligands forming complexes with their cognate targets (ITC) [2,3,4], or arising from direct metabolic activity (IMC) [5,6]. IMC curves exhibit enough easily detectable features to quickly identify growth-promoting or growth-inhibiting factors in defined cultures of microorganisms on a purely qualitative or semi-quantitative basis. These include maximal metabolic power, lag time of growth, and other time-based markers.

Understanding, analyzing, and extracting biologically meaningful information from these features becomes increasingly important as state-of-the-art IMC reveals previously inaccessible details of microbial growth. The field of IMC applications is expected to grow even further with the accumulation of microbe-specific signatures in heat flow curves, on the one hand, and with advanced analysis techniques on the other. This Special Issue collects examples of quantitative IMC analyses which address microbial growth in various contexts. Grütter et al. demonstrate that IMC can be used as a diagnostic tool in urethritis caused by *Neisseria gonorrhoea*, a pathogen which is difficult to culture for diagnostic purposes [7]. With IMC, the microorganism is detectable at concentrations as low as 1 CFU·mL^−1^, revealing both an organism-specific signature in the heat flow curves and its drug susceptibility. Another medical application of IMC is presented by Sigg et al., who show that the metabolic heat generated by *Proteus mirabilis* can serve to determine the efficacy of phages against this urinary tract pathogen [8].

As IMC data are typically recorded under nutrient-limited growth, a novel method to extract the apparent nutrient affinity and maximal growth rates from microbial heat curves is proposed by Fahmy [9]. Here, it is shown that Monod-type growth kinetics are valid far beyond their original restriction to balanced growth. Even the falling phase of nutrient-limited growth can be well described, as shown for *Lactococcus lactis* and *Trypanosoma congolense* cultures. This evaluation method enables extremely sensitive two-dimensional quantification of toxicity. This allowed Oertel et al. to separate the chemical toxicity from detrimental effects of β-radiation on bacterial growth in the presence of radioactive europium [10].

Finally, Doung et al. demonstrate that IMC enables much less error-prone determination of energy utilization patterns of fungi than biomass- and substrate-based estimations [11]. By studying seven fungi with different substrate requirements, the authors show that ecologically relevant “trait-complexes” can be built on metabolic heat curves to predict ecological performance and biomass production by these saprotrophic lignocellulose decomposer fungi.

All authors of this Special Issue are members of the International Society of Biological Calorimetry (ISBC), which brings together researchers with both academic and commercial backgrounds who use IMC in a large variety of applications and share interest in spreading scientific and technical knowledge on the non-invasive observation of live cell metabolism. The idea for this Special Issue originated from many discussions at biennial society meetings. The ISBC held its 21st meeting in Vilnius on 8–10 June 2022 after a 4-year break due to the pandemic. It has served for nearly 50 years as a meeting place for those interested in the measurement of heat and the use of thermodynamics in biology.

What was to become the ISBC began in 1973, when the Swedish instrument manufacturer LKB organized a one-day meeting at Chelsea College, London, UK. The first meeting was called ISMAB: “International Symposium of Microcalorimetric Applications in Biology”, and it was hosted by Anthony Beezer. Most evidence points to the fact that ISMAB became ISBC in 1990 at the seventh meeting, but we include the ISMAB meetings in our ISBC meeting enumeration. ISBC is an informal conference without parallel sessions, typically involving 70 participants, at which all types of calorimetry in systems of biological interest are discussed. The meeting in Vilnius had the typical eclectic mixture of fundamental and applied studies on a broad range of biological systems using different calorimetric techniques in different fields. This was well-phrased in the name given to the eleventh meeting in 1999: “Biothermodynamics: Molecular, Organismal and Ecological”. In Vilnius, we listened to presentations on such diverse issues as the modes of action of specific enzymes, kinetics of the degradation of nano-plastics, oscillating metabolism of fungi, and the relationship between heat engines and the human brain.

The biocalorimetry meetings provide fascinating interaction opportunities for researchers working in physics, chemistry, and biology. The rather informal nature of the ISBC meetings provides plenty of opportunities for interesting discussions. The Special Issue is an outcome of these interactions across disciplines and geographic borders, and will continue to advance an extremely dynamic field. In combination with modern molecular biology methods and genetic engineering, IMC will further contribute to deciphering metabolic mass and energy flows in complex systems at the best of their complexity, i.e., in live organisms. Please visit our website, biocalorimetry.org to follow ISBC until the next meeting in 2024.
